# Effects of Chinese proficiency and illustration types on Chinese reading comprehension among international students: evidence from eye-tracking

**DOI:** 10.3389/fpsyg.2026.1770079

**Published:** 2026-03-23

**Authors:** Qingshan Qiu, Siqin Yin, Wenjie Huang

**Affiliations:** 1 School of Chinese Language and Literature, Hubei University, Wuhan, China; 2 School of Management, Huazhong University of Science and Technology, Wuhan, China

**Keywords:** Chinese as a foreign language, cognitive load, eye-tracking, illustration comprehension, second language reading

## Abstract

**Purpose:**

This research investigates how Chinese proficiency and different types of illustrations affect international students reading comprehension of Chines science and Technology text. Eye tracking technology will be used to clarify the cognitive process involved.

**Methods:**

We conducted a mixed-factorial eye-tracking experiment with a 2 (Chinese proficiency: high vs. low) × 4 (illustration type: representational, explanatory, organizational, transformational) design. We used a Tobii Pro Glasses 2 eye-tracker to record eye movement data, specifically total text-image fixation time, illustration area fixation duration, and fixation count. To analyze the effect of proficiency, illustration type, and their interaction, we applied a Linear Mixed Model (LMM), treating proficiency and illustration type as fixed factors and individual differences as random effects.

**Results:**

The results revealed a significant interaction between Chinese proficiency and illustration type on comprehension scores [*F*(3,82) = 3.25, *p* = 0.035]. Specifically, high-proficiency learners demonstrated better comprehension with explanatory and organizational illustrations. They appeared to leverage the structured features of these semantic integration as evidenced by higher fixation counts on organizational illustrations (28.07). Conversely, low-proficiency learners relied more on representational illustrations to reduce cognitive load. They also exhibited significantly increased fixation counts and durations when processing transformational illustrations (total fixation duration: 325.62 s), suggesting that their limited language capacity led to cognitive resource competition and integration difficulties.

**Conclusion:**

This study confirms a proficiency-illustration matching mechanism in Science and Technology Chinese reading. Key findings indicate that: (1) high-proficiency learners benefit most from explanatory and organizational illustrations, while low-proficiency learners rely on representational illustrations to reduce cognitive load; (2) transformational illustrations pose higher processing demands for low-proficiency learners, reflected in prolonged fixation durations; and (3) eye-movement patterns reveal distinct, proficiency-dependent processing strategies. The results underscore the need for aligning illustration types with learners’ Chinese proficiency to optimize comprehension and cognitive efficiency in academic second-language contexts.

## 1 Introduction

The classification of illustration types commonly used in psychological reading research (referred to as “illustration types” in this paper) is based on the Mayer (1993), functional categorization of images which mainly includes five types (Levin et al., 1987; Winn, 1993). Decorative illustrations such as flowers, borders, and other elements do not add information to the text but only beautify it. Representational illustrations depict events, characters, concepts, and other elements described in the text, such as character portraits of or relevant buildings. Organizational illustrations integrate and organize textual information to clarify the structural and organizational relationships within the text, for instance maps and mind maps. Explanatory illustrations can be combined with text and employ concrete methods, such as metaphors and analogies, to illustrate the essence and internal connections of things or concepts. For example, bees symbolize diligence and effort, or experimental results can be illustrated with annotated text. Transformational illustrations can reshape readers’ cognitive patterns by translating abstract concepts into concrete mental images. This process encourages readers to connect new information with prior knowledge and experiences, sparking emotional engagement and stimulating the imagination through visuals such as cartoons and data driven charts.

Textual illustrations are important media for multimodal cognition, serving not only as auxiliary visual tools but also as core elements of knowledge construction. Illustrations can compensate for the lack of context in pure text through the dual coding of cultural symbols (e.g., Lindauer and Kennedy, 1974), moreover they can also help address the issues faced by students with mathematical learning difficulties (Zhang et al., 2022). Illustrations can significantly improve the efficiency of knowledge memory, and their hierarchical visual design can optimize the expression of the internal structure of information, playing a key role in cognitive enhancement (Duchastel and Waller, 1979). The combination of text and images can significantly improve the accuracy of reading comprehension among middle school students (Tao et al., 2003).

Cognitive load is a multidimensional structural element that describes the load on the cognitive system when learners process specific tasks (Wang and Fu, 2021). Physiological measurement methods, which assess changes in physiological indicators generated by subjects during task performance, can be used to measure cognitive load (Wierwille and Eggemeier, 1993), as can measurements of brain activity (Wickens et al., 2013). Eye-trackers are also widely used to measure cognitive load, with related research gradually advancing to the level of cognitive mechanisms. Based on cognitive load theory, Mayer (1993) argued that the functional classification of illustrations directly affects learners’ information processing efficiency where the organizational illustrations reduce intrinsic cognitive load through hierarchical structures, while transformational illustrations promote cross-modal knowledge transfer through metaphorical design. Studies have confirmed that various eye movement indicators reflect cognitive load and integration efficiency during vocabulary processing. This allows for analysis of the differences between initial vocabulary exposure and subsequent semantic integration. These findings are also applicable to research on cognitive load in text-image processing (Pellicer-Sánchez, 2016). Studies indicate that readers exhibit individual differences in eye movements. Furthermore, cognitive load in multimedia learning is regulated by three factors including task complexity, which directly affects cognitive resource requirements; individual learner differences, such as cognitive ability, prior knowledge, and strategy automation, which alter resource allocation; and the organization and presentation of content, which triggers changes in load by influencing information processing paths (Wang and Fu, 2021). The information capacity of illustrated textbooks is positively correlated with the breadth of visual attention (Levie et al., 1982). Individuals with high cognitive ability process scientific illustrations more efficiently (Hnanus et al., 1999). Ding et al. (2023) demonstrated that regression path duration and total fixation duration are effective indicators of the cognitive difficulty associated with subsequent semantic integration. Similarly, Hou and Tu (2023) found that low-proficiency learners exhibit increased fixation counts when processing abstract illustrations, potentially reflecting an imbalance in cognitive resource allocation resulting from limited language skills. Further supporting this line of inquiry, Adam et al. (2021) analyzed regressive and corrective eye movements in 120 participants during multi-line text reading to investigate the impact of spelling and reading ability. Chinese texts in science and technology are highly specialized, exhibiting logical structures and dense terminologies far exceeding that of general Chinese. Successfully navigating these texts requires learners to utilize both organizational illustrations to reinforce semantic networks (Yan et al., 2018) and to simultaneously integrate terminologies with illustrations, interpreting abstract concepts through illustration. This presents international students with limited language proficiency with two key challenges: overcoming language barriers and synchronously integrating professional knowledge (Paivio, 1986). Furthermore, it strains the cross-modal resource allocation abilities of students with lower proficiency (Sweller, 1988). However, the dual coding of linguistic and visual information has been shown to improve memory and comprehension efficiency (Paivio, 1986), and the pictographic nature of Chinese characters may increase learners’ reliance on visual representations (Yu, 2011). This study builds upon existing research, which has several limitations. First, most experiments rely on materials in the participants’ native language, leaving unclear how second language learners dynamically allocate cross-linguistic cognitive resources (Cerda et al., 2019). Second, the cognitive synergy between Chinese characters and scientific illustrations remains underexplored (Yu, 2011; Yan et al., 2018). Third, there is a scarcity of research on the interaction between illustration types and learner proficiency, particularly in the context of second language learners reading scientific and technical Chinese. For example, Yan et al. (2018) identified an interaction between illustration types and academic performance but did not investigate how second language ability regulates visual processing. Similarly, Liu et al. (2002) distinguished between static and dynamic illustrations without analyzing multimodal integration efficiency in relation to language ability. Finally, while Cerda et al. (2019) found that second language learners exhibit lower cross-modal integration efficiency compared to native speakers, but the mechanism by which Chinese proficiency moderates the cognitive benefits of different illustration types requires further investigation. Current science and Technology text books in chines primarily utilize representational, organizational, explanatory, and transformational illustrations, with decorative illustrations appearing infrequently. This study designed an eye-tracking experiment, focused on international science and engineering students reading scientific and technical text, using above mentioned four common illustration types. This study analyzes the cognitive interaction between Chinese proficiency and illustration types to reveal how second language learners uniquely process cross-modal information. By exploring the dynamic synergy between visual aids and language ability in Science and Technology Chinese reading, this research provides an empirical basis for designing textbook illustrations enabling students to efficiently build professional knowledge. The study explores the following questions: (1) How do Chinese proficiency and illustration types impact reading efficiency and depth of comprehension? (2) Do eye movement patterns differ between learners with varying Chines proficiency levels when processing four illustrations types? (3) How do illustration types moderate the relationship between Chinese proficiency and cognitive load?

## 2 Methods

### 2.1 Participants and experimental design

This study included 32 international science and engineering students (15 males and 17 females) from 20 countries, representing 17 majors at a university in Wuhan. The participant ranged in age from 18 to 38 years (mean age = of 22.84 years), all had normal or corrected-to-normal vision. They had studied Chinese for 10 to 48 months, and all had passed the reading section of the HSK (Hanyu Shuiping Kaoshi), with scores above 60. The students were divided into two groups based on their proficiency: a high-proficiency group (HSK Level 5–6) and a low-proficiency group (HSK Level 3–4), each group counting 16 participants.

The study used a 2 (Chinese proficiency: high vs. low) × 4 (illustration types) mixed-factorial experimental design. Chinese proficiency (high vs. low) was the between-subjects factor, and illustration type was the within-subjects factor. Prior to the experiment, all participants completed a standard 9-point binocular calibration for the Tobii Pro Glasses 2 (calibration error < 0.5° of visual angle; re-calibrated if failed), with the device adjusted to fit individual head shapes for stable wearing. *A priori* power analysis for the mixed design was conducted via G*Power 3.1, with the interaction effect as the primary index, *α* = 0.05 and medium effect size *f* = 0.25 set as parameters. The results indicated a minimum sample size of 28 was required to achieve a statistical power of 1-*β* = 0.85, and our sample size of 32 thus meets the sufficient power requirement for the main research questions. The interaction effect in this study was a small-to-medium effect (*η*^2^ = 0.08), and the sample size was not further expanded due to practical constraints of eye-tracking experiments (e.g., time-consuming data collection and strict experimental environment requirements).

### 2.2 Experimental tools

In this experiment, a Tobii Pro Glasses 2 wearable eye-tracker (100 Hz sampling rate) recorded participants’ eye movements. The eye-tracker employed corneal reflection and dark pupil tracking to collect binocular data. AOI mapping was implemented using Tobii Pro Lab’s AOI Editor, where illustration AOIs were defined by pixel coordinates of their outer contours to realize precise matching of eye-movement data to the illustration area. Tobii Pro Lab (x64) software and R software were used to analyze the collected eye movement data. To investigate the influence of Chinese proficiency and illustration type on international students’ Chinese reading comprehension, we employed a Linear Mixed Model. Given the research focus on the overall cognitive investment in illustration areas during scientific text reading, we initially selected core eye-tracking metrics reflecting the total processing effort of text-image integration, with the aim of providing a clear and foundational analysis of the proficiency-illustration matching mechanism.

### 2.3 Experimental materials

The experimental articles were developed with reference to the International Chinese Education Chinese Proficiency Standards. Each article contained 400 Chinese characters, with accompanying exercises totaling approximately 110 characters. The illustrations, with a uniform physical size of 18 cm^2^ (size standardization) and printed in color, were positioned on the right side of the text. To eliminate the interference of visual complexity on eye-tracking results, three experts in applied psychology rated the visual complexity of the four types of illustrations using a 5-point Likert scale (1 = extremely simple, 5 = extremely complex). The results showed no significant difference in visual complexity among the four types of illustrations (*M* ± *SD* = 2.1 ± 0.3, *p* > 0.05), ensuring the consistency of visual cognitive load across illustration types. Moreover, five senior international Chinese teaching experts rated the information matching degree between each illustration type and the corresponding text with a 5-point Likert scale (1 = extremely low matching, 5 = extremely high matching), and statistical analysis confirmed no significant difference in informational content equivalence among the four types (*p* > 0.05). In addition, the Area of Interest (AOI) for each illustration was demarcated at the pixel level along the outer contour of the illustrations; two experimenters independently completed the AOI demarcation, and the intraclass correlation coefficient (ICC) of the demarcation results was greater than 0.95, which guaranteed the reliability and objectivity of AOI definition and eye-tracking data extraction. The text was wrapped around the illustrations on the left, and the text, illustrations, and exercises all appeared on the same page. To balance the materials, a Latin square design was employed. Each article was paired with four different illustration types, creating a 4×4 Latin square of experimental articles and illustration types, and resulting in 16 unique sets of experimental materials. The presentation order of these materials was randomized for each participant to avoid order effects. Following each article, participants answered five true-false questions to assess their comprehension (maximum score: 5). Of the five questions, three were illustration-relevant items that required integration of illustration information for accurate answering, and two were text-based basic items. The internal consistency reliability of the 50 comprehension questions (10 articles × 5 items) was verified via Cronbach’s *α* coefficient (*α* = 0.78), and a pre-experiment confirmed all items had a discrimination index of 0.3–0.7, ensuring the sensitivity and reliability of the comprehension assessment.

The text and true-false questions maintained consistent fluency and difficulty across all materials. Twenty Chinese university students rated fluency using a Likert scale, where 5 indicated “very fluent.” The mean fluency score for the four sets of materials was 4.745 (*SD* = 0.1525). Separately 10 international Chinese teachers rated the difficulty of the materials, where 5 indicated “very difficult.” The mean difficulty score was 2.9275 (*SD* = 0.245), which was moderate and suitable for the participating international students with HSK Level 3–6 proficiency.

### 2.4 Eye-tracking metrics definition

In line with the research focus on exploring the cognitive processing differences of international students with different Chinese proficiency levels when reading scientific and technological texts with different illustration types, three core eye-tracking metrics were selected for analysis. All metrics were extracted from the Tobii Pro Lab software, with the Area of Interest (AOI) defined as the entire illustration region (standardized as described in 2.3). The selection of metrics was closely tied to the research questions and the measurement of cognitive load in text-image integration, with specific definitions and cognitive implications as follows:

1. Total Text-Image Fixation Time: The total duration of all fixations on the combined text and illustration areas during the entire reading process, reflecting the overall cognitive effort invested by learners in processing the multimodal text. Longer total fixation time is generally associated with higher cognitive load caused by text comprehension or illustration integration difficulties (Ding et al., 2023; Hou and Tu, 2023).
2. Illustration Area Fixation Duration: The cumulative fixation time on the illustration AOI, representing the specific cognitive attention allocated by learners to visual aids. This metric reflects the degree of reliance on illustrations for reading comprehension, and its variation is closely related to the cognitive load generated by illustration type processing (Yan et al., 2018).
3. Illustration Area Fixation Count: The total number of fixations on the illustration AOI, an indicator of the frequency of cognitive processing of visual information. Higher fixation counts indicate repeated processing of illustration content, which is a typical behavioral manifestation of increased cognitive load when learners encounter difficulties in cross-modal integration (Pellicer-Sánchez, 2016).

The selection of the above metrics was based on the primary research goal of revealing the proficiency-illustration matching mechanism in Science and Technology Chinese reading. By focusing on the overall and specific cognitive investment in illustration areas, the study aims to provide a straightforward and empirical basis for the dynamic adaptation of learners with different proficiencies to various illustration types, laying a foundational framework for subsequent in-depth research with more detailed eye-tracking metrics.

## 3 Data analysis and results

### 3.1 Descriptive statistics and correlation analysis

A two-way mixed analysis of variance revealed significant strategic differences between the two proficiency groups in the cognitive processing of illustration types. All eye-tracking AOI indicators were naturally controlled for physical size interference due to the uniform 18 cm^2^ size of all experimental illustrations, and no additional numerical correction was required.

The results indicated a significant main effect of Chinese proficiency, *F*(1,30) = 8.42, *p* < 0.05, *η^2^* = 0.22, and illustration type,: *F* (3,90) = 5.15, *p* < 0.05, *η^2^* = 0.18. The interaction effect, however, was not significant, *F* (3,90) = 1.89, *p* = 0.14, *η^2^* = 0.05 (Table 1). The high proficiency group demonstrated significantly better comprehension scores and fixation efficiency (fixation count to duration ratio) compared to the low-proficiency group. Explanatory illustrations and transformational illustrations yielded the highest comprehension scores, while organizational illustrations and representational illustrations yielded the lowest scores, respectively. The high-proficiency group recorded a fixation count of 28.07 and total text-image fixation time of 280.88 s for organizational illustrations; the low-proficiency group recorded a total text-image fixation time of 325.62 s and fixation count of 16.62 for transformational illustrations, and a total text-image fixation time of 302.83 s for representational illustrations, with a fixation count of 11.00 and total text-image fixation time of 244.13 s for organizational illustrations.

**Table 1:** Descriptive statistical results of variables (*M ± SD*).

Proficiency group	Illustration type	Comprehension score	Total text-image time	Illustration area duration	Illustration area fixation count
High proficiency	Representational	3.53 ± 1.51	259.55 ± 92.88	3.81 ± 4.17	10.47 ± 7.74
	Explanatory	4.33 ± 0.72	271.60 ± 102.24	6.69 ± 8.76	15.27 ± 15.27
	Organizational	4.14 ± 1.23	280.88 ± 111.15	9.26 ± 8.29	28.07 ± 15.95
	Transformational	4.36 ± 0.84	260.64 ± 94.70	6.22 ± 5.23	19.93 ± 18.40
Low proficiency	Representational	3.29 ± 1.20	302.83 ± 148.52	2.64 ± 2.20	10.50 ± 8.26
	Explanatory	2.88 ± 1.26	290.30 ± 145.29	2.55 ± 1.96	10.50 ± 5.76
	Organizational	2.81 ± 1.11	244.13 ± 166.65	3.42 ± 2.61	11.00 ± 6.19
	Transformational	3.25 ± 1.18	325.62 ± 117.00	5.12 ± 5.00	16.62 ± 12.15

### 3.2 Linear mixed model analysis

Given the nested and non-independent characteristics of eye-tracking data (with multiple observations per participant) and the limitations of the traditional mixed analysis of variance (ANOVA) in accounting for individual differences among participants, we further adopted a Linear Mixed Model (LMM) for in-depth statistical analysis. The preliminary two-way mixed ANOVA only provided exploratory descriptive results of the main and interaction effects, without controlling for the random variation caused by individual differences in eye movement and reading comprehension, which may lead to biased judgments on the actual effect of the variables. LMM, by incorporating individual differences as random effects and treating Chinese proficiency and illustration type as fixed effects, can more accurately capture the true interaction between the independent variables and their predictive effects on the dependent variables, and is the gold standard statistical method for analyzing eye-tracking and repeated measures data in cognitive and second language acquisition research (Meteyard and Davies, 2020; Staub, 2021). The following sections detail the construction, validation and fitting results of the LMM model.

#### 3.2.1 Model construction

In this experiment, the dependent variables included comprehension scores, total text-image fixation time (abbreviated as “total text-image time”), illustration area fixation duration (abbreviated as “illustration area duration”), and illustration area fixation count (abbreviated as “illustration area count”). Chinese proficiency (a binary variable), illustration type (a four-category variable), and their interaction effect were included in the model as fixed effects to explore the differential impacts of illustration types on groups with different proficiency levels. Random effects were used to control biases caused by individual differences among participants. The mathematical expression of the linear mixed model is as follows:


$$\begin{array}{l} Y_{\mathrm{ij}}=\beta_{0}+\beta_{1}\cdot \text{Proficiency Grou}p_{i}\;+\beta_{2}\cdot \text{Illustration}\;\mathrm{Typ}e_{j}\\ +\beta_{3}\cdot (\text{Proficiency Grou}p_{i}\times \text{Illustration}\;\mathrm{Typ}e_{j})+(1|\text{Subjec}t_{i})+\epsilon_{\mathrm{ij}} \end{array}$$

Where $Y_{\mathrm{ij}}$ represents the eye movement data of the i-th participant under the j-th illustration type; $\beta_{0}\;$ is the intercept term; $\;\beta_{1}$ , $\beta_{2}$ , and $\beta_{3}$ are the fixed effect coefficients for Chinese proficiency, illustration type, and their interaction, respectively; $\;(1|\text{Subjec}t_{i})$ is the random effect term to control for individual differences among students; and $\epsilon_{\mathrm{ij}}$ is the error term.

#### 3.2.2 Model validation

The study used linear mixed models to test hypotheses for the four dependent variables. The results showed that the models effectively explained the sources of variation in the dependent variables, with both statistical robustness and explanatory power.

**Figure 1:**
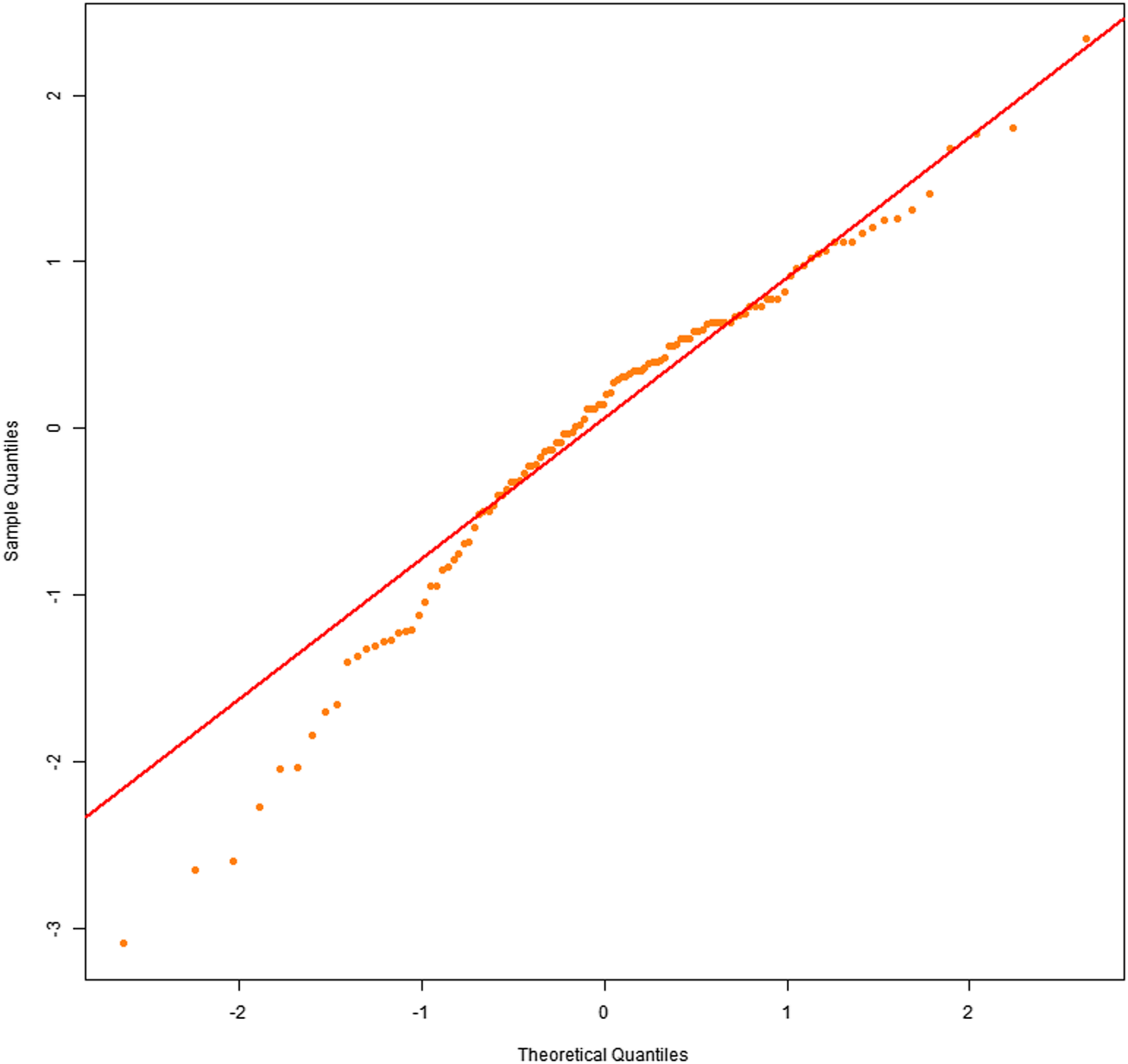
Q–Q plot of comprehension scores.

**Figure 2:**
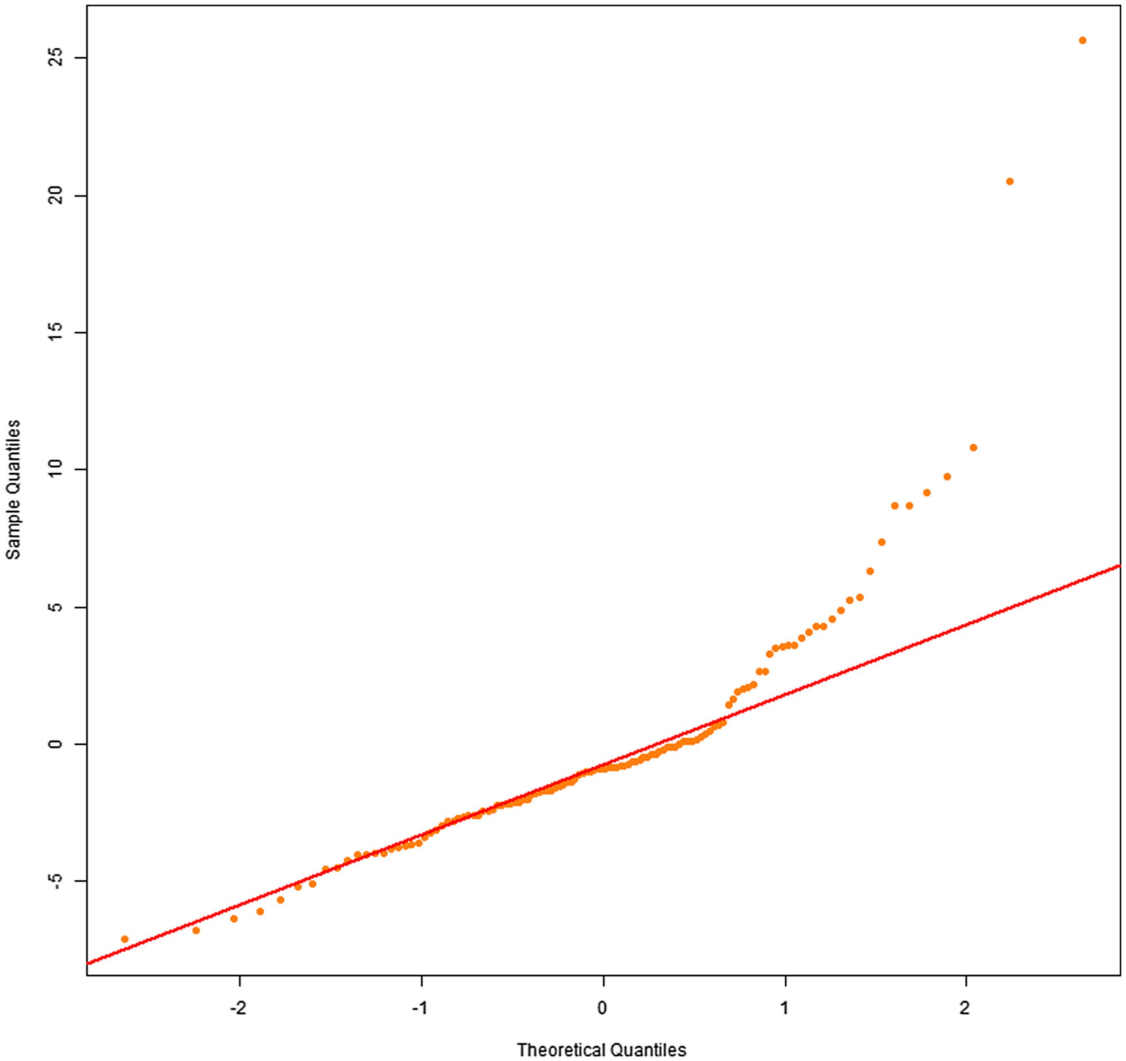
Q–Q plot of illustration area duration.

**Figure 3:**
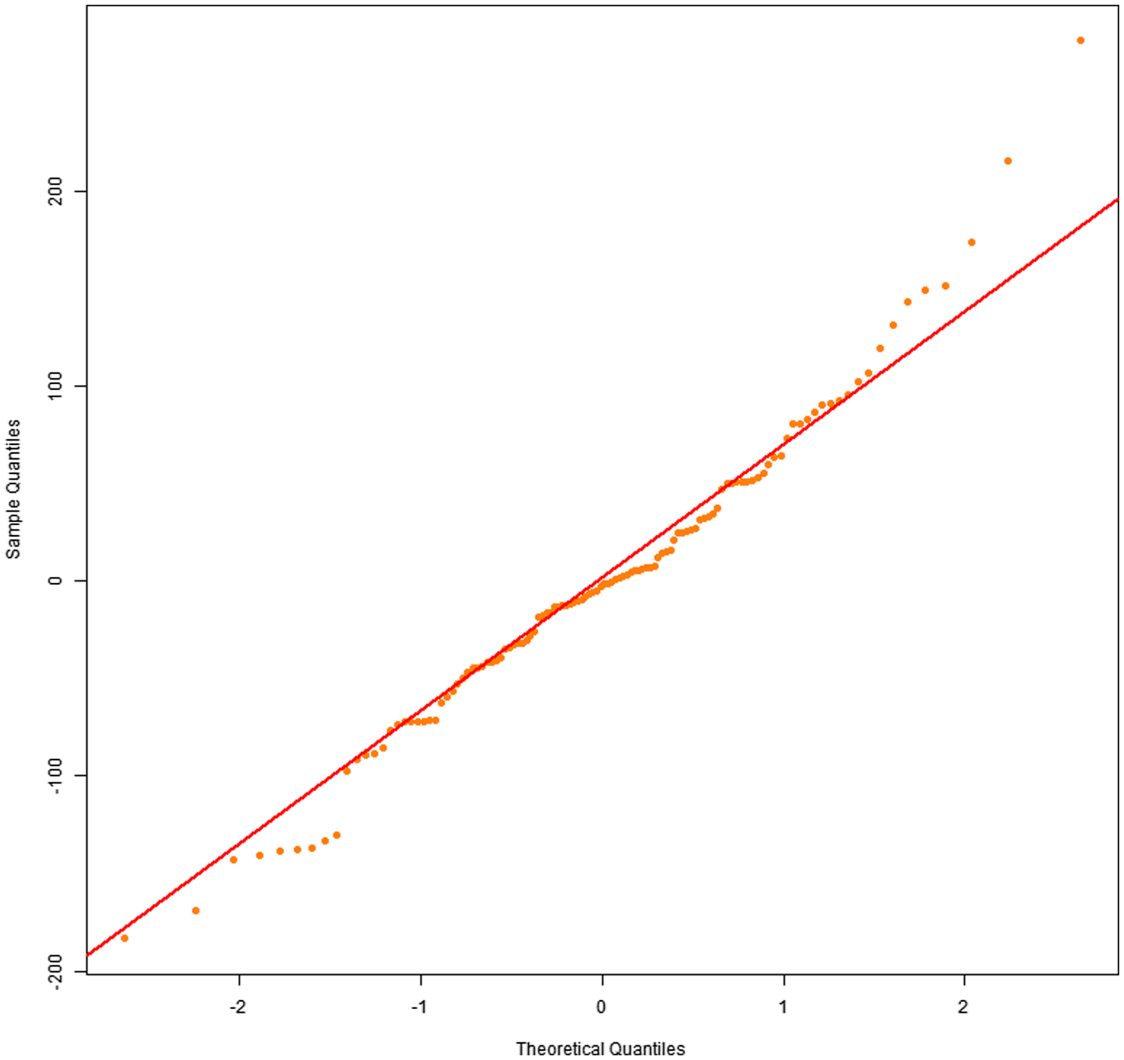
Q–Q plot of total text-image time.

**Figure 4:**
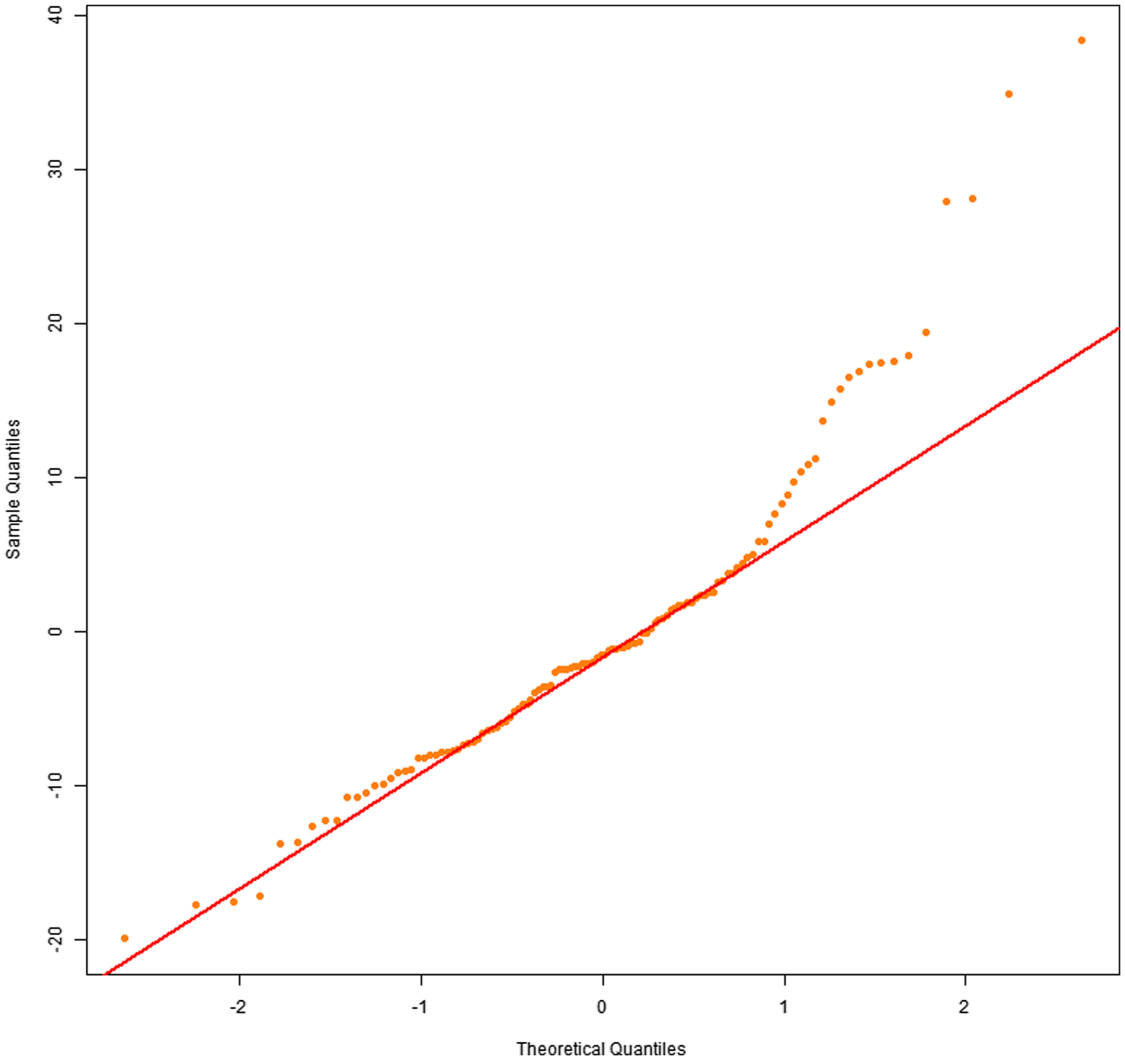
Q–Q plot of illustration area count.

Q–Q plots indicated that the residuals for all four dependent variables generally met the normality assumption. Specifically, Figures 1, 2 showed residual points closely aligned with the diagonal of the normal distribution, with only minor deviations in the tails. Figure 3 residuals were highly consistent with a normal distribution in the middle range but showed significant tail deviations. Figure 4 displayed data points primarily distributed along the diagonal. Overall, the model residuals were approximately normally distributed.

**Figure 5:**
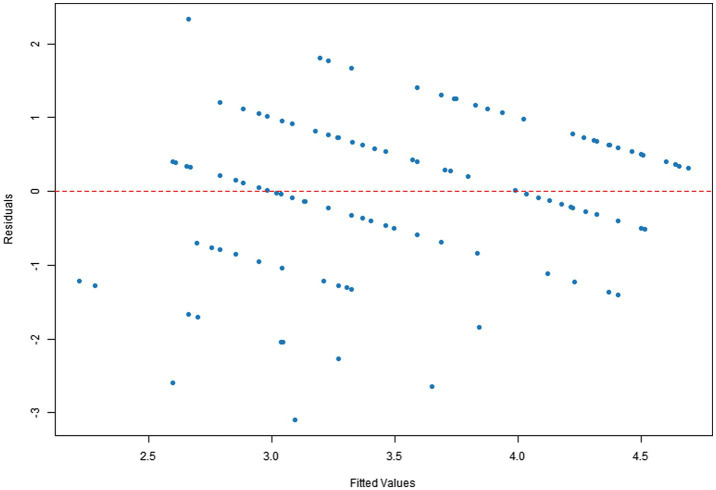
Plot of comprehension score residuals vs. fitted values.

**Figure 6:**
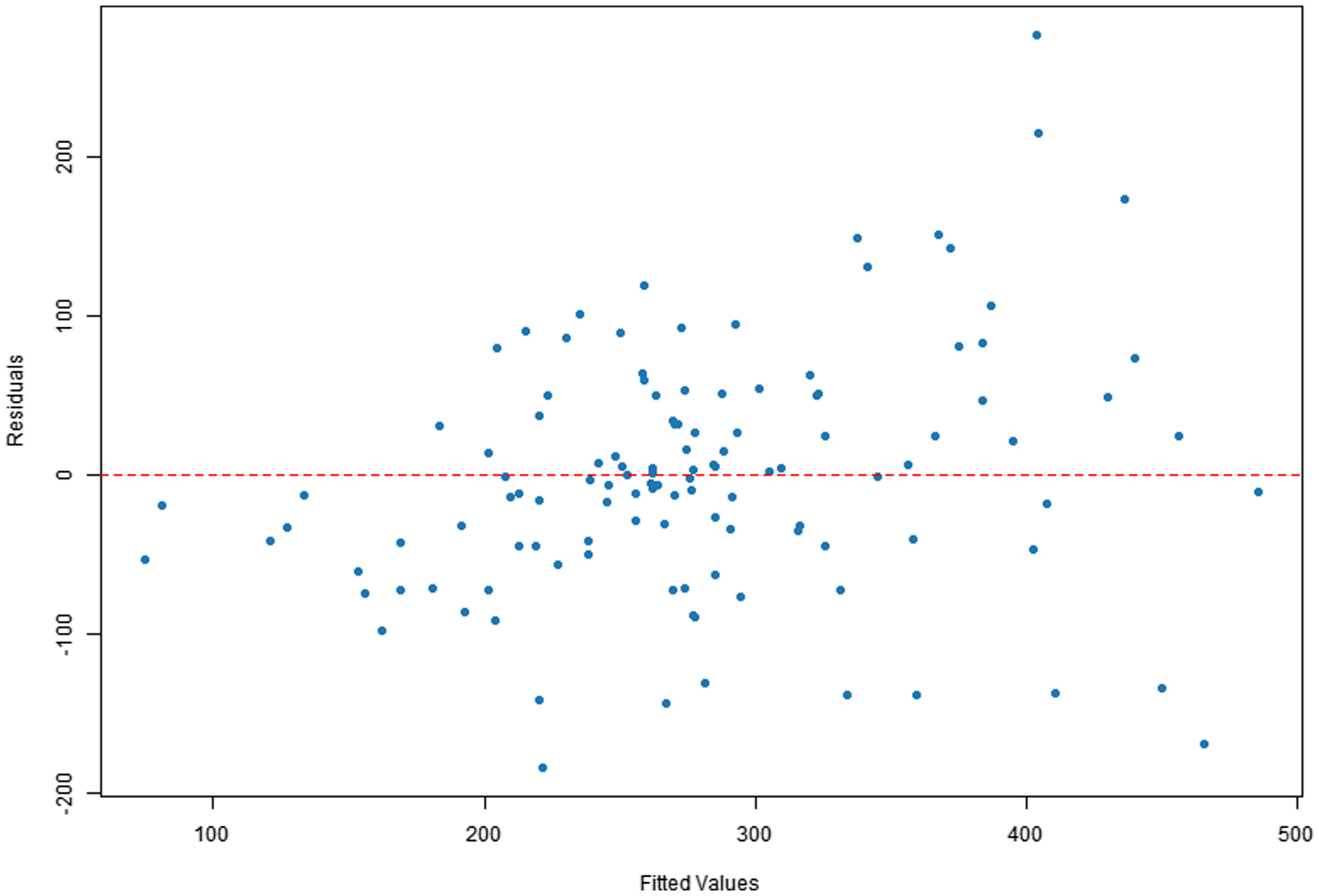
Plot of total text-image time residuals vs. fitted values.

**Figure 7:**
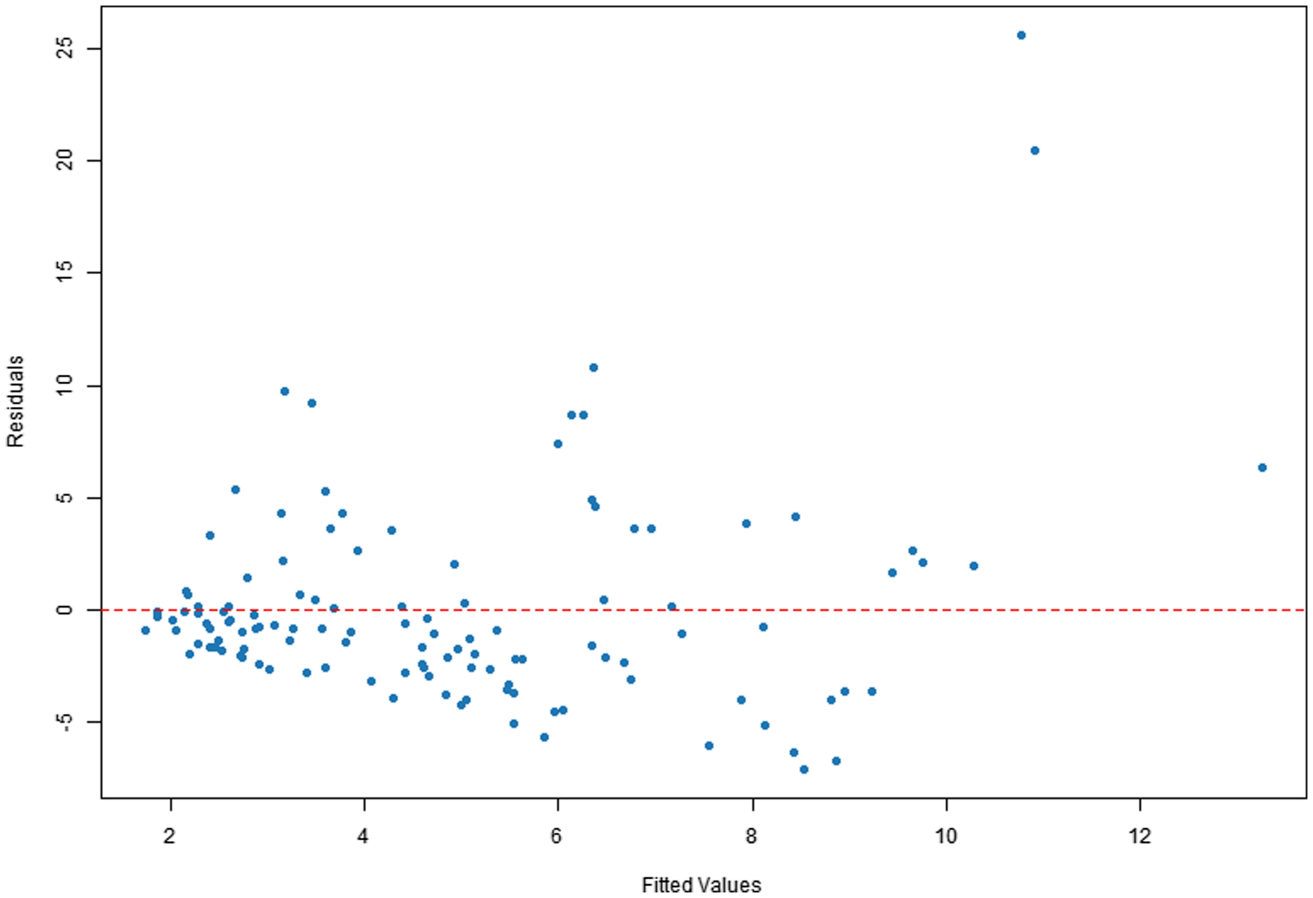
Plot of illustration area duration residuals vs. fitted values.

**Figure 8:**
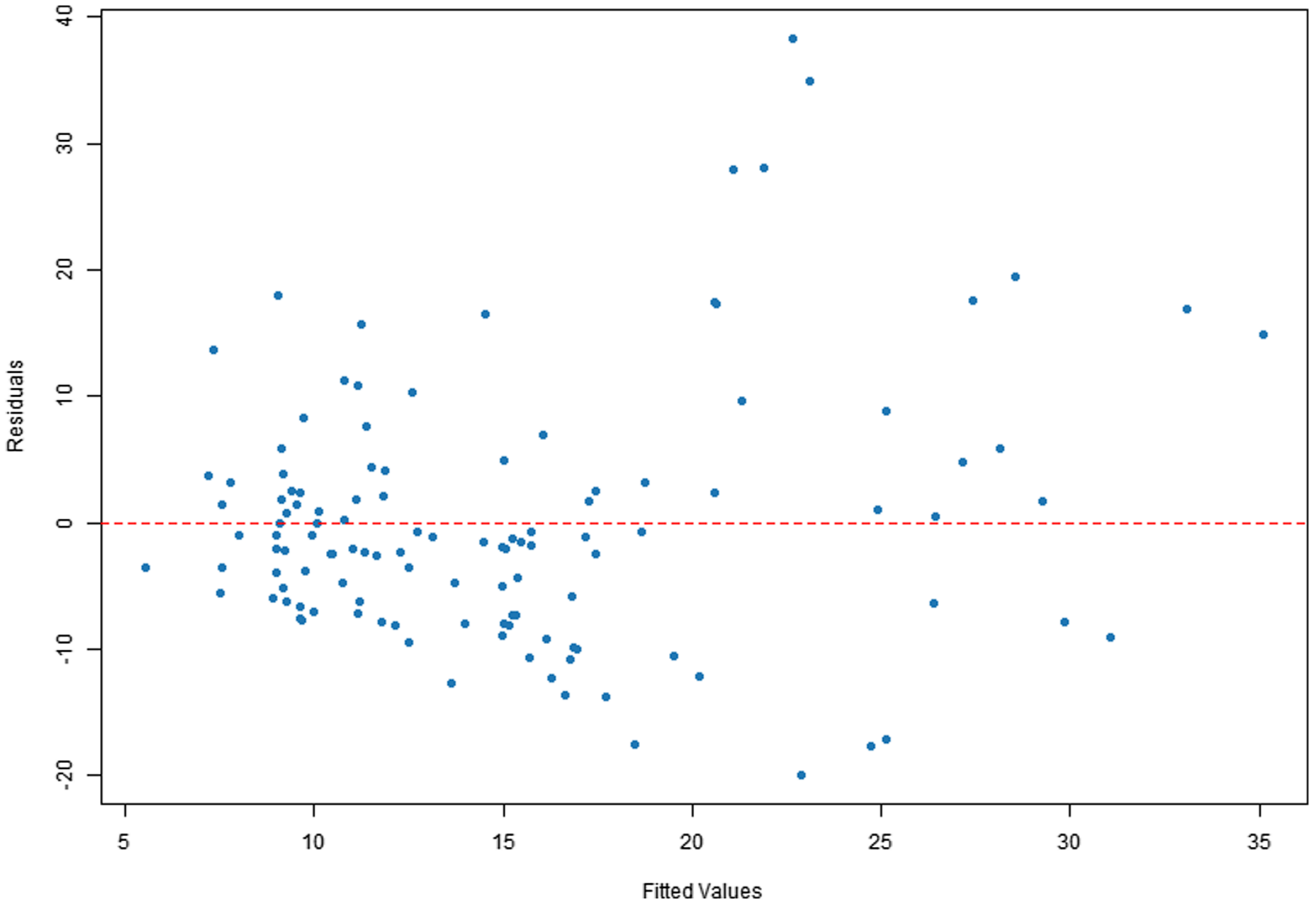
Plot of illustration area count residuals vs. fitted values.

Scatter plots of residuals versus fitted values indicated that the model assumptions were generally met for all four dependent variables. Specifically, Figures 5, 6 show a random distribution of data points with no significant trends, supporting the assumptions of independence and homoscedasticity. Figure 7 shows a generally reasonable residual distribution, with random fluctuations accounting for noise in the high-fitted value region. While Figure 8 shows local dispersion in the high-fitted region, the overall data still meets the homoscedasticity assumption.

The standard deviation of the subject intercept for comprehension scores was 0.42 (*p* < 0.001; see Table 2), indicating considerable individual variation. Furthermore, the proportion of variance explained by random effects for each dependent variable (e.g., 18.7% for total text-image time) corroborated the importance of accounting for participant heterogeneity. Model comparisons, based on AIC values, showed that models with random effects exhibited significantly lower AIC values than fixed-effect models (e.g., a decrease from 1045.67 to 1023.45 for comprehension scores).

**Table 2:** Analysis results of random effects for dependent variables.

Dependent variable	Std. dev. of subject intercepts	Std. dev. of residuals
Comprehension score	0.42	1.08
Total text-image time	0.50	2.13
Illustration area duration	0.39	1.66
Illustration area count	0.46	1.23

#### 3.2.3 Model fitting results

Linear mixed model results indicated that Chinese proficiency and illustration type had significant effects on multiple dependent variables, and interaction effects also played important roles in specific indicators.

##### 3.2.3.1 Comprehension scores

The main effects of Chinese proficiency and illustration type were not significant [Chinese proficiency: *F*(1,82) = 0.30, *p* = 0.587; illustration type: *F* (3,82) = 1.14, *p* = 0.338], indicating that neither the improvement in language ability alone nor differences in illustration types had a direct significant impact on comprehension scores (Table 3). However, their interaction effect was significant [*F*(3,82) = 3.25, *p* = 0.035, $\eta^{2}$ = 0.08], revealing conditional differences in the support provided by illustration types to high and low proficiency groups. Specifically, explanatory illustration provided more significant support to the high-proficiency group (between-group difference: 0.52 points, *p* = 0.008, after Bonferroni correction) with a moderate effect size (*d* = 0.45), while the between-group differences for organizational illustration and representational illustration gradually narrowed. The latter, due to their intuitive design, provided more balanced support to both groups (difference: 0.07 points, *p* = 0.62).

**Table 3:** Linear mixed model results for comprehension scores.

Fixed effects	Estimate	Std. error (*SE*)	Degrees of freedom (*df*)	*t*-value	*p*-value
Intercept	3.29	0.31	108.466	10.67	<0.001
Chinese Proficiency (High vs. Low)	0.23	0.43	107.851	0.55	0.587
Illustration Type (Transformational vs. Representational)	−0.42	0.40	82.737	−1.06	0.293
Illustration Type (Explanatory vs. Representational)	−0.05	0.40	82.737	−0.11	0.911
Illustration Type (Organizational vs. Representational)	−0.48	0.40	82.737	−1.22	0.227
Chinese Proficiency × Explanatory Diagram	1.20	0.56	83.061	2.14	0.035
Chinese Proficiency × Transformational Diagram	0.86	0.57	83.721	1.52	0.133
Chinese Proficiency × Organizational Diagram	1.11	0.56	82.196	1.97	0.052

**Figure 9:**
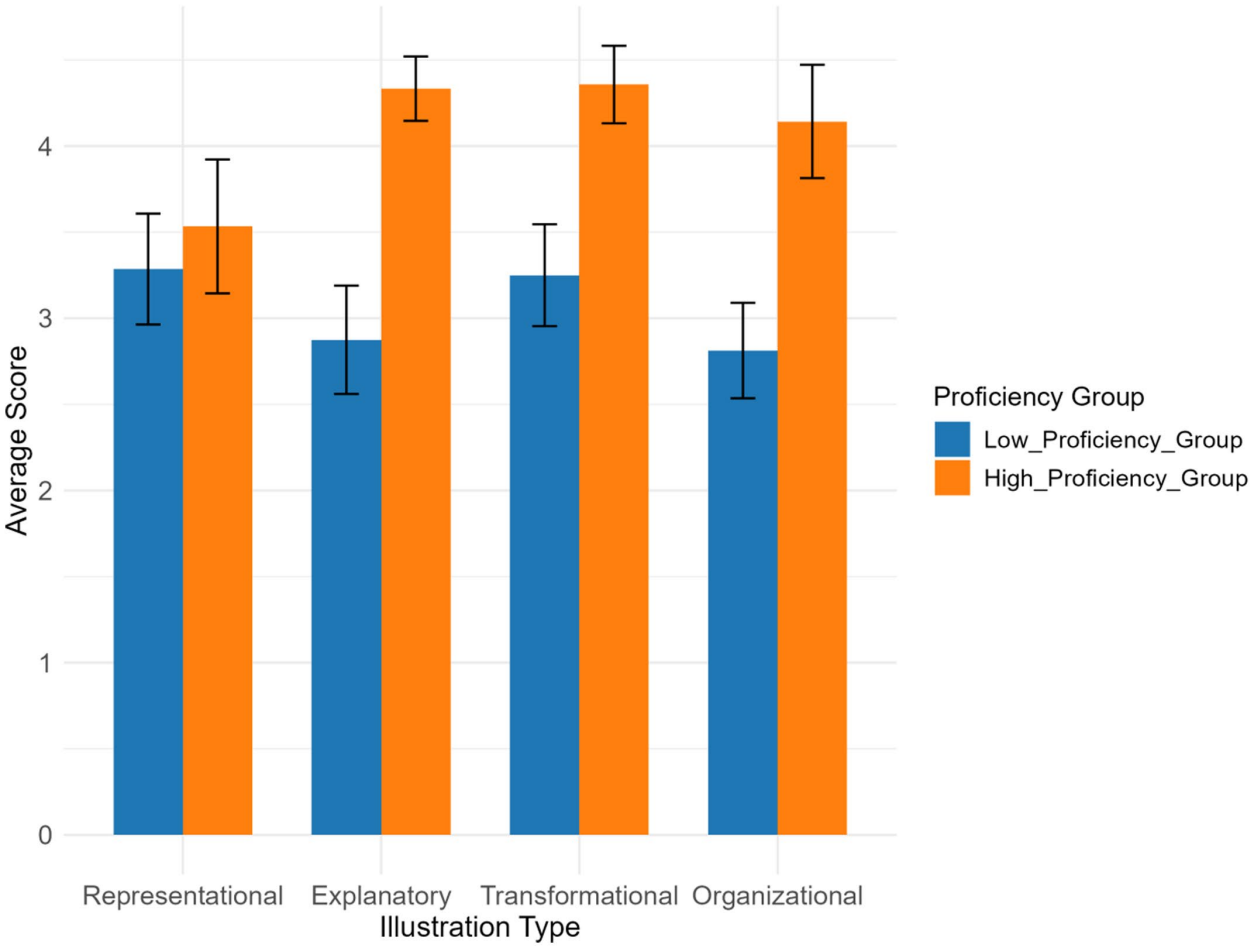
Comprehension scores across illustration types and Chinese proficiency groups.

The high-proficiency group achieved significantly higher comprehension scores than the low-proficiency group across all illustration types (Figure 9). The score gaps between the two groups were larger for explanatory illustrations and organizational illustrations, but smaller for representational illustrations.

##### 3.2.3.2 Total text-image time

The differences in total text-image time between the two groups was only 40.07 s. The low-proficiency group generally spent more total text-image time than the high-proficiency group across all illustration types, indicating that illustrations type had no significant impact on total text-image time (Table 4). Neither the main effect of Chinese proficiency (*p* = 0.389) nor the main effect of illustration type on total text-image time was significant, and the interaction effect was also non-significant.

**Table 4:** Linear mixed model results for total text-image time.

Fixed effects	Estimate	Std. error (*SE*)	Degrees of freedom (*df*)	*t*-value	*p*-value
Intercept	296.57	33.01	72.31	8.99	<0.001
Chinese Proficiency (High vs. Low)	−40.067	46.20	70.44	−0.87	0.389
Illustration Type (Transformational vs. Representational)	−6.266	32.90	82.45	−0.19	0.849
Illustration Type (Explanatory vs. Representational)	29.045	32.90	82.45	0.88	0.380
Illustration Type (Organizational vs. Representational)	−52.44	32.90	82.45	−1.59	0.115
Chinese Proficiency × Explanatory Diagram	14.497	46.56	82.86	0.31	0.756
Chinese Proficiency × Transformational Diagram	−31.879	47.09	83.14	−0.68	0.502
Chinese Proficiency × Organizational Diagram	68.337	46.80	82.25	1.46	0.148

**Figure 10:**
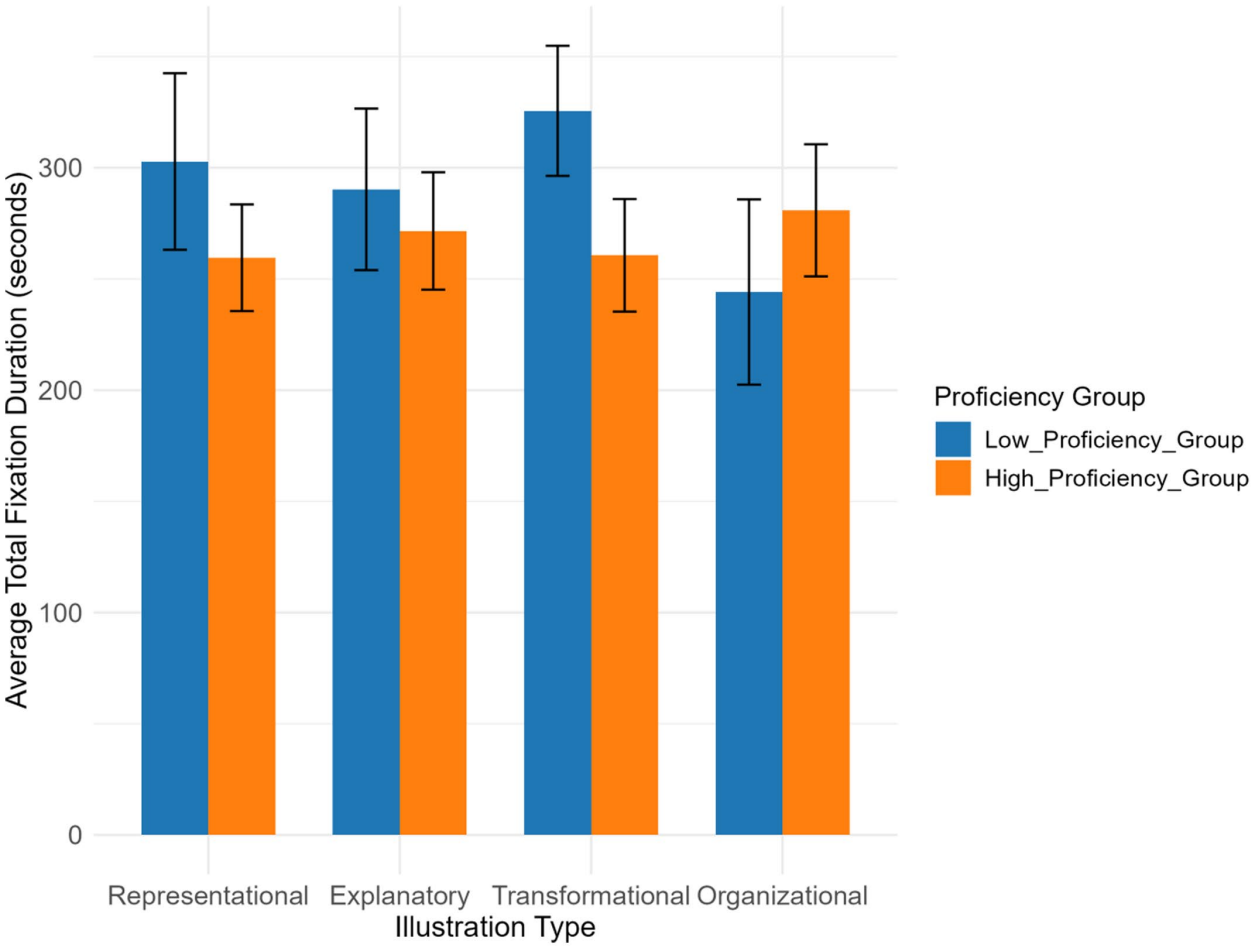
Total fixation duration across illustration types and Chinese proficiency groups.

The low-proficiency group spent more fixation time on representational illustration (Figure 10). The high-proficiency group had longer fixation time on organizational and transformational illustrations, and the two groups showed little difference in fixation time on explanatory illustration.

##### 3.2.3.3 Illustration area duration

The effect estimate for Chinese proficiency was 1.060 (*p* = 0.592), and differences between various illustration types and the baseline (representational illustration) were not significant (e.g., transformational illustration: *p* = 0.946; explanatory illustration: *p* = 0.186) (see Table 5). In addition, interaction effect analysis indicated that the interaction between Chinese proficiency and organizational illustration approached the significance threshold (estimate = 4.626, *p* = 0.079). All other interaction terms were non-significant (all *p* > 0.05). The intercept term of the model was marginally significant (*p* = 0.061).

**Table 5:** Linear mixed model results for illustration area duration.

Fixed effects	Estimate	Std. error (*SE*)	Degrees of freedom (*df*)	*t*-value	*p*-value
Intercept	2.68	1.42	109.23	1.89	0.061
Chinese Proficiency (High vs. Low)	1.06	1.97	108.73	0.54	0.592
Illustration Type (Transformational vs. Representational)	−0.12	1.83	84.26	−0.07	0.946
Illustration Type (Explanatory vs. Representational)	2.44	1.83	84.26	1.33	0.186
Illustration Type (Organizational vs. Representational)	0.74	1.83	84.26	0.40	0.688
Chinese Proficiency × Explanatory Diagram	3.01	2.58	84.55	1.16	0.247
Chinese Proficiency × Transformational Diagram	−0.04	2.61	85.21	−0.02	0.986
Chinese Proficiency × Organizational Diagram	4.63	2.65	83.72	1.78	0.079

**Figure 11:**
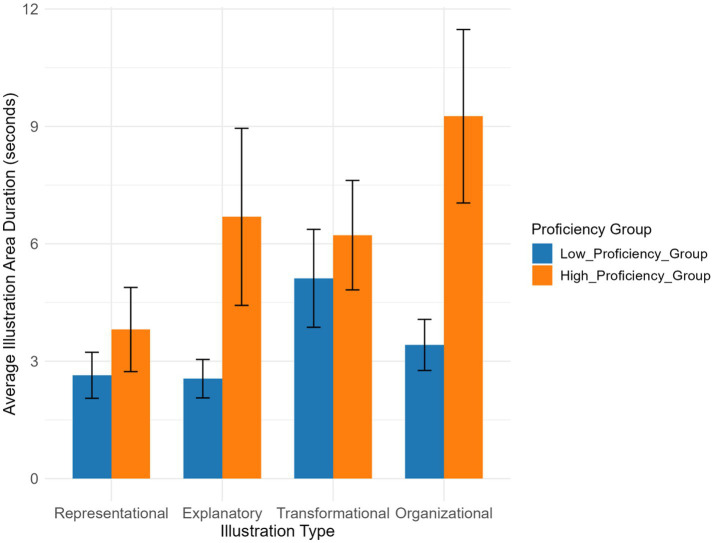
Illustration area duration across illustration types and Chinese proficiency groups.

The high-proficiency group generally spent longer fixation durations on all illustration types than the low-proficiency group (Figure 11). Specifically, the high-proficiency group had the longest fixation duration on organizational illustration and the shortest on representational illustration, while the low-proficiency group spent relatively longer on transformational illustration. Overall, the fixation durations of the high-proficiency group on illustrations were more strongly influenced by illustration type, whereas differences among the low-proficiency group were relatively smaller.

##### 3.2.3.4 Illustration area count

The main effect of Chinese proficiency (*p* = 0.942) and the main effect of illustration type (all *p* > 0.05) on illustration area count were non-significant. The interaction effect between Chinese proficiency and organizational illustration was significant (*β* = 16.934, *p* = 0.004) (see Table 6). All other interaction effects were non-significant (all *p* > 0.05).

**Table 6:** Linear mixed model results for illustration area count.

Fixed effects	Estimate	Std. error (*SE*)	Degrees of freedom (*df*)	*t*-value	*p*-value
Intercept	10.56	3.19	108.138	3.32	<0.001
Chinese Proficiency (High vs. Low)	−0.32	4.43	107.485	−0.07	0.942
Illustration Type (Transformational vs. Representational)	6.06	4.06	84.289	1.49	0.139
Illustration Type (Explanatory vs. Representational)	−0.06	4.06	84.289	−0.02	0.988
Illustration Type (Organizational vs. Representational)	0.44	4.06	84.289	0.11	0.914
Chinese Proficiency × Explanatory Diagram	0.01	5.74	84.616	0.87	0.385
Chinese Proficiency × Transformational Diagram	3.36	5.79	85.238	0.58	0.564
Chinese Proficiency × Organizational Diagram	16.93	5.78	83.781	2.93	0.004

**Figure 12:**
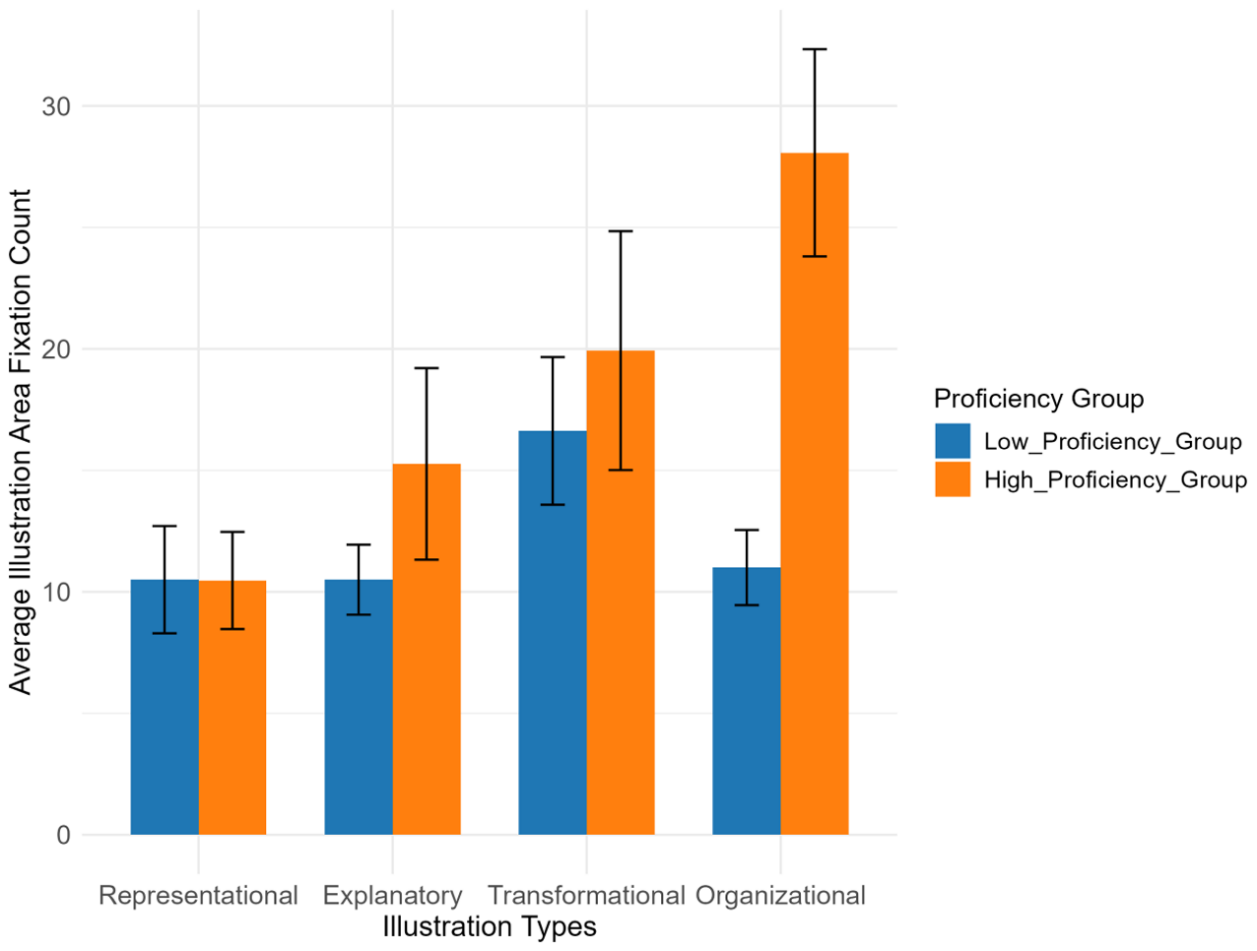
Illustration area fixation count across illustration types and Chinese proficiency groups.

The high-proficiency group had more fixations than the low-proficiency group across most illustration types (Figure 12), with the most significant between-group difference observed for organizational illustration and greater data variability. In contrast, between-group differences were smaller for representational, explanatory, and transformational illustrations.

### 3.3 Summary

Chinese proficiency and illustration type jointly influenced international students’ reading comprehension performance and eye movement processing patterns of text-image materials. The high-proficiency group obtained higher comprehension scores when processing illustration types requiring in-depth semantic analysis and logical integration, including explanatory and organizational illustrations. The low-proficiency group showed relatively better comprehension performance with representational illustrations, and exhibited longer fixation time and higher fixation count when processing transformational illustrations.

Eye movement indicators showed consistent group differences: the high-proficiency group presented more targeted fixation patterns on illustration areas with high information value (e.g., organizational illustrations), while the low-proficiency group showed more scattered fixation distribution and longer total text-image fixation time across all illustration types. The interaction between Chinese proficiency and illustration type was significant in key indicators including comprehension scores and illustration area fixation count, reflecting the differential adaptation of learners with different Chinese proficiency levels to various illustration types.

## 4 Discussion

### 4.1 Consistency of the study and theoretical expansion

This study confirms the cognitive enhancement effect of illustrations in multimodal learning (Lindauer and Kennedy, 1974; Duchastel and Waller, 1979) and reveals the regulatory mechanism of language ability on cross-modal integration paths.

These findings also enrich the empirical evidence base for contemporary multimodal learning analytics in second language contexts, providing objective eye-tracking indicators for quantifying cognitive processing patterns in text-image multimodal reading. The observed proficiency-dependent eye movement characteristics further complement the theoretical framework of eye-tracking guided multimodal learning research, offering actionable insights for the design of evidence-based multimodal reading interventions for L2 learners.

#### 4.1.1 Deepening and boundary correction of dual coding theory

Dual coding theory emphasizes that the collaborative coding of language and visual channels can improve information processing efficiency (e.g., Paivio, 1986). This study found that high-proficiency learners can significantly optimize comprehension efficiency through in-depth integration of dual coding such as the mapping between the logical framework of organizational diagrams and text semantics. This is consistent with Tao et al.’s (2003) findings on the synergistic effect of text and images. However, due to insufficient automation of language decoding, low-proficiency learners may experience cross-modal resource competition caused by dual coding (Sweller, 1988), leading to cognitive overload when processing abstract illustration types (e.g., transformational diagrams). These findings revises the applicable boundary of dual coding theory in second language scenarios, indicating that language ability is a core regulatory variable for cross-modal integration efficiency, rather than a universal advantage simply relying on visual assistance.

#### 4.1.2 Dynamic interpretation of cognitive load theory

Cognitive load theory emphasizes the dynamic balance between task complexity and individual ability (Sweller, 1988). This study is consistent with the core hypothesis of the theory where the low-proficiency learners show significant increases in scattered fixations and regression frequency when processing high-abstract illustration types, indicating that their intrinsic cognitive load threshold is exceeded; high-proficiency learners reduce extraneous load through structured diagrams (e.g., organizational diagrams), achieving efficient reconstruction of semantic networks. These finding supports cognitive load theory from the dimension of language ability regulation, i.e., the “load threshold” does not exist statically but changes dynamically with language proficiency, being deeply bound to the learner’s information processing stage.

#### 4.1.3 Cross-modal extension of second language acquisition research

Existing studies have shown that the cross-modal integration efficiency of second language learners is generally lower than that of native speakers (e.g., Cerda et al., 2019). This study found that differences in Chinese proficiency can explain the significant differentiation in illustrations type processing efficiency. For example, low-proficiency learners show systematic lag in illustration types requiring logical reasoning (e.g., organizational diagrams), with their comprehension scores being less than 70% of those of high-proficiency learners. This gap far exceeds the typical effect size of illustrations type influence in native language research (e.g., Yan et al., 2018), indicating that the effectiveness of illustrations in second language scenarios highly depends on the “bandwidth support” of language ability. The research results resonate with Yan et al.’s (2018) study on the processing of topological structures of Chinese characters, revealing the compensatory and limiting characteristics of second language learners’ visual dependence.

### 4.2 “Proficiency-modal-cognition” three-dimensional interaction mechanism

This study proposes a “proficiency-modal-cognition” three-dimensional interaction mechanism, which can systematically explain the dynamic relationship between Chinese proficiency, illustration types, and cognitive processing paths.

#### 4.2.1 Language ability as the regulatory hub of cognitive bandwidth

High-proficiency language learners, having developed an automated language system, free up working memory. This allow them to engage in top-down semantic integration, utilizing schema-driven approaches such as hierarchical analysis of organizational diagrams. This processing aligns with Mayer (1993) cognitive construction theory, but highlights the critical role of language automation in cross-modal resource allocation. Conversely, low-proficiency learners expand significant cognitive resources on language decoding. This forces them to rely on visual compensation strategies, using concrete elements of representational diagrams to sustain basic comprehension. This differentiation dictates cognitive bandwidth, offering a theoretical foundation for research into individual differences in second language reading.

#### 4.2.2 Dual-path differentiation of illustration type functions

Schema-driven path: High proficient learners leverage organizational and transformational diagrams to activate prior knowledge networks in long-term memory, creating a concept transfer-semantic verification” loop. The intensive nature of this approach characterized by concentrated fixation, suggests that strong language skills representational constrains of diagrams, transforming them into advanced cognitive tools.

Visual compensation path: Learner with weaker language skills, facing language barriers employ a “local anchoring-linear concatenation” strategy relying on item-by-item correspondence in representational diagrams. Their dispersed fixation pattern reveals a disconnect in cross-modal integration. However, this approach reduce immediate cognitive load, it hinder the development of deep semantic networks, supporting Liu et al. (2002) findings on the effectiveness of static illustrations.

#### 4.2.3 Dynamic adaptability of cognitive load threshold

This study revealed that low-proficiency learners have a cognitive load threshold, primarily dominated by a single mode (unimodal), and their ability to integrate information across multiple modes (cross-modal integration) is easily overwhelmed by abstract illustrations. In contrast, high-proficiency learners exhibit a more balanced cognitive load threshold across multiple modes (multimodal), allowing them to dynamically regulate load through strategic adjustments, such as selective fixations. These findings provide empirical support for Adam et al. (2021) research on individual differences and demonstrate a positive correlation between “threshold elasticity” and language ability.

### 4.3 Synergistic effects of multi-level mechanisms

The differentiation phenomena found in the study can be attributed to the synergistic effects of the following multi-level mechanisms.

#### 4.3.1 Language-dependent allocation of working memory resources

High-proficiency learners allocate more working memory resources to higher-order integration processes like logical reasoning. In contrast, low-proficiency learners must dedicate more resources to language decoding. This difference in allocation allows high proficiency learner to process text and image in parallel, while lower-proficiency learners experience a processing bottleneck characterized by “decoding priority-integration lag” (Pellicer-Sánchez, 2016). These findings broaden the scope of the “hierarchical bottleneck” theory in second language reading by considering a cross-modal perspective.

#### 4.3.2 Threshold effect of language automation degree

Once Chinese proficiency reaches a sufficient level (e.g., HSK Level 5), the language decoding becomes automated, freeing up cognitive resources for learner to integrate the logical structure of diagrams with the meaning of the text. However, below this level, learner rely on serial processing, which can hinder information retention (e.g., through repeated regressions when viewing transformational diagrams). This difference explains the distinct effect of academic level and language ability on the effectiveness of different types of illustration (Yao et al., 2018).

#### 4.3.3 Dual influences of L1 cognitive inertia

The pictographic nature of the Chinese character system (Yu, 2011) may increase learners reliance on visual representations. However, understanding the abstract logic of scientific diagrams such as circuit diagrams, requires moving beyond L1 cognitive habits. Low-proficiency learners may experience processing delays due to cognitive conflicts between the topological representation of Chines choristers characters and the rules governing the scientific diagrams. In contrast, higher-proficiency learners can leverage their language skills to quickly internalize diagram rules. These findings provides new empirical evidence for cross-linguistic transfer theory.

### 4.4 From micro-design to reconstruction of educational ecology

This study transcends the conventional separation of text and image by establishing a three-dimensional “proficiency-modality-cognition” interaction mechanism. This mechanism fosters a paradigm shift in second language reading theory, moving from static description to dynamic regulation. Ultimately, this contributes to the development of a precise, inclusive and sustainable international Chinese education ecosystem.

#### 4.4.1 Dynamic adaptation principles for illustration design

Beginner textbooks should emphasize representational and simplified organizational diagrams. This approach minimize cross-modal load by using through concrete examples and avoiding cognitive overload caused by abstract diagrams. Intermediate textbooks should incorporate explanatory diagrams that help learners connect abstract concepts with concrete representations. They should also introduce semi-structured transformational diagrams to develop cross-modal transfer skills. Advanced textbooks should prioritize organizational diagrams alongside highly metaphorical transformational diagrams to encourage structured integration and cross-domain application of professional knowledge.

This proficiency-adaptive illustration design aligns with the signaling and segmenting effects in L2 scaffolded instruction: for low-proficiency learners, a segmented presentation of text and illustrations is recommended to reduce extraneous cognitive load, while high-proficiency learners benefit from an illustration-first presentation with text verification, which enhances active semantic integration and logical reasoning.

#### 4.4.2 Differentiated intervention strategies in classroom teaching

Develop a task sequence that progresses with the complexity of illustrations. Evaluate learning effectiveness by considering both comprehension depth and processing efficiency. Use eye tracking metrics to real-time identify students’ cognitive load in real time, differentiating between learners experiencing “resource competition” and those with “strategy deficiencies.” For students struggling with resources competition, implementa stepwise text-image stepwise presentation approach: first strengthen language decoding skills, then gradually introduce diagrammatic logic. For students with strategy deficiencies, provide “metacognitive training” to guide them their attention to the critical elements within the diagrams.

#### 4.4.3 Empowering teaching with multimodal cognitive tools

Teachers should integrate multimodal cognitive tools into their teaching to improve students ability to diagnose diagram by assessing their logical integration level based on eye moment, behavior; enhance their hierarchical design by developing an “illustration type-task-ability” matching matrix for the dynamic, gradient design of teaching materials; and strengthen technology integration ability by operating eye trackers and adaptive learning platforms. These tools can automatically generate illustration type combinations based on learners’ Chinese proficiency profiles and dynamically adjust abstraction degree and information density. By Identifying processing bottlenecks (e.g., scattered fixations in transformational diagrams) through real-time eye tracking, the tools can trigger dynamic simplification of diagrams or provide additional language annotations. Finally, these tools can generate individual “cognitive strength-weakness” reports based on the correlation between fixation patterns and comprehension scores to facilitate precision teaching.

With the development of educational technology, AI-generated multimodal supports can further optimize this personalized teaching model: AI systems can be trained on the proficiency-illustration matching mechanism identified in this study to automatically generate and adjust illustration types, information density and presentation modes according to learners’ real-time Chinese proficiency, reading performance and eye movement characteristics, realizing intelligent and dynamic multimodal scaffolding for Science and Technology Chinese reading. This AI-integrated approach provides a new development direction for the digital transformation of international Chinese education multimodal teaching.

### 4.5 Research limitations and future directions

This study has certain limitations that need to be acknowledged, and these limitations also point to directions for future research. First, the eye-tracking metrics employed in this study are focused on the overall cognitive investment in illustration areas (e.g., total fixation time, fixation count), and core metrics closely related to fine-grained cognitive load and cross-modal integration were not included, such as mean fixation duration, regression frequency/duration, time-to-first fixation, saccade amplitude, and pupil diameter. These metrics can reflect the dynamic cognitive processing process (e.g., initial attention allocation, semantic integration difficulty, emotional arousal) in more detail (Staub, 2021; Wang et al., 2023), and their exclusion may limit the in-depth interpretation of the cognitive mechanisms underlying the proficiency-illustration interaction. Second, the sample size of this study is relatively small (*N* = 32), although power analysis has confirmed that the sample size meets the basic statistical requirements for the main research questions, the generalizability of the findings needs to be further verified with a larger and more diverse sample (e.g., international students from different language backgrounds and academic majors). Third, the experimental materials are limited to scientific and technological texts of a fixed length (400 Chinese characters), and the applicability of the proficiency-illustration matching mechanism to texts of different lengths, difficulty levels, and genres (e.g., narrative and expository) remains to be explored.

Future research can be expanded in the following aspects: First, incorporate fine-grained eye-tracking metrics (e.g., mean fixation duration, regressions, pupil diameter) to explore the dynamic cognitive processing differences of learners with different Chinese proficiency levels when processing various illustration types, and reveal the micro-mechanisms of cross-modal resource allocation and cognitive load regulation in more detail. Second, expand the sample size and diversity to verify the generalizability of the proficiency-illustration matching mechanism, and further explore the moderating effects of variables such as L1 background, academic major, and learning strategies. Third, diversify experimental materials by including texts of different lengths, difficulty levels, and genres, and explore the boundary conditions of the proficiency-illustration matching mechanism in different reading contexts. Fourth, combine multiple research methods (e.g., interviews, think-aloud protocols) with eye-tracking technology to conduct a mixed-methods study, which can complement the objective eye movement data with subjective cognitive experience, and provide a more comprehensive understanding of the cognitive process of international students reading Science and Technology Chinese with illustrations. Finally, based on the findings of this study, develop and validate adaptive illustration design systems for international Chinese education, and explore the practical effect of proficiency-matched illustrations in real teaching scenarios.

## 5 Conclusion

This study employed an eye-tracking experiment to investigate how Chinese proficiency and illustration types interactively shape the reading comprehension of Science and Technology Chinese among international students. The central finding supports a proficiency-illustration matching mechanism, wherein the cognitive utility of an illustration is not absolute but moderated by the learner’s linguistic capacity.

The results revealed that high-proficiency learners benefited more from semantically integrated illustrations—such as explanatory and organizational types—which facilitated deeper text-image integration. In contrast, low-proficiency learners relied on representational illustrations to reduce cognitive load, while transformational illustrations imposed processing demands that often exceeded their available cognitive resources, as reflected in prolonged fixation durations.

These findings extend established cognitive theories to second-language academic contexts. They delineate a boundary condition for Dual Coding Theory by highlighting that efficient verbal-visual synergy presupposes sufficient language proficiency. Similarly, they elaborate Cognitive Load Theory by showing that the same illustration may function as germane load for advanced learners but as extraneous load for beginners.

Pedagogically, this research advocates for a proficiency-sensitive approach to designing multimodal materials for Science and Technology Chinese. Educators and material developers should tailor illustration types to learners’ language levels: representational and structured organizational illustrations for lower-proficiency learners, and progressively more abstract and integrative illustrations for advanced learners, to foster both comprehension and higher-order reasoning skills.

In sum, this study underscores that visual aids in L2 disciplinary reading are not universally effective; their impact is dynamically regulated by the learner’s language proficiency. Aligning illustration types with learners’ linguistic readiness can thus create more cognitively efficient and equitable pathways to academic literacy in Chinese.

## Data Availability

The datasets presented in this study can be found in online repositories. The names of the repository/repositories and accession number(s) can be found: https://dataverse.harvard.edu/.
